# One possible mechanism for eddy distribution in zonal current with meridional shear

**DOI:** 10.1038/s41598-018-28465-z

**Published:** 2018-07-04

**Authors:** YunLong Shi, DeZhou Yang, XingRu Feng, JiFeng Qi, HongWei Yang, BaoShu Yin

**Affiliations:** 1 School of Marine Sciences, Nanjing University of Information Science and Technology, Nanjing, China; 2 Key Laboratory of Ocean Circulation and Waves, Institute of Oceanology, Chinese Academy of Sciences, Qingdao, China; 3 University of Chinese Academy of Sciences, Beijing, China; 4 Function Laboratory for Ocean Dynamics and Climate, Qingdao National Laboratory for Marine Science and Technology, Qingdao, China; 5 College of Mathematics and System Science, Shandong University of Science and Technology, Qingdao, China

## Abstract

Oceanic mesoscale eddies are common, especially in areas where zonal currents with meridional shear exists. The nonlinear effects complicate the analysis of mesoscale eddy dynamics. This study proposes a solitary (eddy) solution based on an asymptotic expansion of the nonlinear potential vorticity equation with a constant meridional shear of zonal current. This solution reveals several important consequences. For example, cyclonic (anticyclonic) eddies can be generated by the negative (positive) shear of the zonal current. Furthermore, the meridional structure of an eddy is asymmetrical, and the center of a cyclonic (anticyclonic) eddy tilts poleward (equatorward). Eddy width is inversely proportional to shear intensity. Eddy phase speed is proportional to shear intensity and the wave amplitude, and their spatial distribution show band-like pattern as they propagate westward. This nonlinear solitary solution is an extension of classical linear Rossby theory. Moreover, these findings could be applied to other areas with similar zonal current shear.

## Introduction

Mesoscale eddies are ubiquitous in the ocean, with typical horizontal scales on the order of 100 kilometers and timescales on the order of a month. Mesoscale eddies play a key role in the transport and mixing of momentum and tracers across the World Ocean. Satellite observations of sea surface height (SSH) have enabled visualization of the westward phase propagation of SSH anomalies. It has been revealed that the phase speeds of isolated abnormal SSHs that are faster than expected based on classic mid-latitude Rossby wave theory^1^. This finding has triggered a number of theoretical works exploring the enhancement with consideration of background flow^2^, bottom topography^3^, other factors^4–7^. Killworth and Blundell^8,9^ provided a comprehensive theory (including both background flow and topography effects) that showed a large-scale bottom slope is insufficient to explain the observed rate of propagation. Aoki *et al*.^10^ analyzed the output of a high-resolution ocean general circulation model, and argued that the dominant factor enhancing the phase speed is bottom pressure decoupling related to rough bottom topography, while north of 30°N, the background flow makes a strong contribution to the enhancement of phase speed. However, some of the observed Rossby wave features cannot be interpreted using classical Rossby wave theory. Subsequent observations of high-resolution SSH fields, constructed by merging the measurements of two simultaneously operating altimeters, have revealed that westward-propagating signals can be identified as nonlinear eddies^11,12^. Previous theoretical research has focused mainly on linear models or numerical simulations^13^ because of the difficulty in solving nonlinear problems.

The nonlinear term reflects important physical phenomena. Long^14^ found that solitary waves are possible in a fluid system confined by rigid boundaries, and in certain atmospheric motions. Boyd^15^ applied the multiple scales method to the primitive equations to show that long, small amplitude Rossby waves are governed by either the Korteweg-de Vries (KdV) equation or modified KdV (mKdV) equation. Helfrich *et al*.^16,17^ developed an asymptotic time-dependent theory for coherent structures on a marginally stable baroclinic flow. Yang *at el*.^18^ discussed the interaction of algebraic solitary Rossby waves with topography and atmospheric blocking. Hodyss and Nathan^19^ studied the dynamics of solitary Rossby waves in a meridionally sheared, zonally varying jet flows, and showed that the zonally varying background flow yielded three general classes of behavior: reflection, transmission, or trapping. They show the oscillatory decay, creation and steady state of solitary Rossby waves, when there is zonally varying jet flow. The KdV equation also has been obtained by Benney^20^, Redekopp^21^, Yamagata^22^, and Warn^23^. Based on KdV equation, the authors investigated the spatial structure and propagation characteristics of solitary waves, and point out that the background flow shear is a necessary condition for the existence of solitary waves.

Despite recent progress, how the varying background flows influence the phase speed, wave width and the symmetry of solitary Rossby wave, however, remains to be addressed. Based on the reduced-gravity potential vorticity equation, and using scale transformations and the perturbation expansions method^14,15,24^, a nonlinear model and its corresponding asymptotic solution are derived in this paper. By using the asymptotic solution, the influence of background flow shear is discussed in detail. Furthermore, we examine some typical solutions and compare them with classical Rossby wave theory in the interpretation of the observed westward-propagating features.

## Observed SSH anomalies

**Figure 1:**
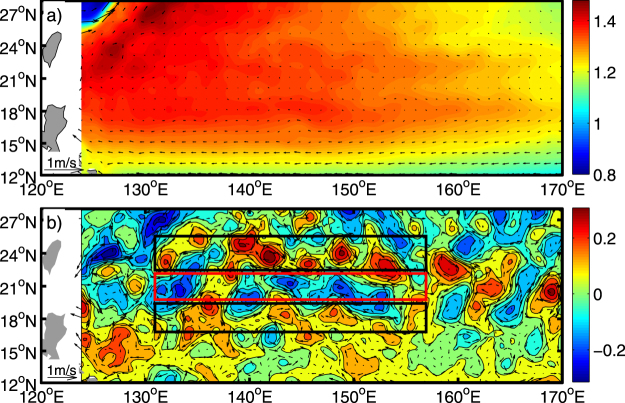
Characteristics of sea level anomalies in the western Pacific Ocean: (a) mean absolute dynamic topography (ADT) and geostrophic current averaged over the period 1993–2012, and (b) sea level anomaly (SLA) and geostrophic current on May 8, 2007. The altimeter products with a daily temporal resolution and 1/4° × 1/4° spatial resolution were produced by DUACS and distributed by AVISO (ftp://ftp.aviso.altimetry.fr/).

**Figure 2:**
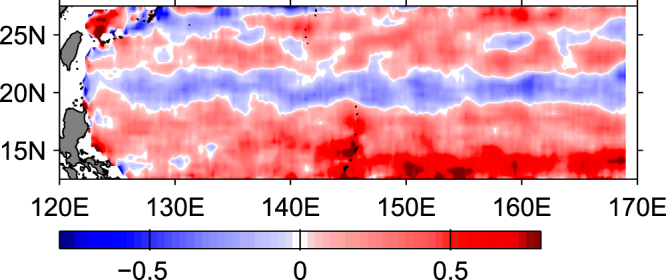
Spatial distributions of eddy polarity (blue for cyclonical eddies and red for anticyclonical eddies)^25^. (With permission from Yang G).

Figure 1 shows the typical characteristics of sea level anomalies in the western Pacific Ocean (12–26°N, 130–180°W). In this paper, we treat isolated anomalies (mesoscale eddies) as solitary Rossby waves, in order to compare them with classic Rossby waves. These isolated anomalies are located mainly between 17–26°N, where the meridional shear of the background zonal current exists and the number of anticyclonic eddies is larger than cyclonic eddies. A noticeable feature is that the meridional distribution of mesoscale eddies shows a band-like structure^25^, where cyclonic eddies might exist in one latitude band and anticyclonic eddies exist in the adjacent latitude band (see Fig. 2). This “band-like” pattern in the ocean have been a subject of numerous studies starting from Galperin *et al*.^26^ and Maximenko *et al*.^27^. Using baroclinic quasigeostrophic models and altimetry sea level anomaly data, Chen *et al*.^28^ suggested that cyclonic and anticyclonic eddies emerge at alternate latitude bands, and confirmed the existence of eddy trains. Cheng *et al*.^29^ investigated the statistical characteristics of mesoscale eddies in the North Pacific, and they suggested that the Subtropical Counter Current (STCC) zone is one of the regions with most eddy activity. Qiu *et al*.^30^ pointed out that in the STCC area, both submesoscale and mesoscale eddies are influenced by baroclinic instability, and show seasonal variation.

Moreover it has been reported that the westward-propagating wave (SSH anomalies) has some meridional asymmetry^31^, it does not show Rossby wave dispersion^32^, and it demonstrates a westward increase in amplitude and phase speed^1^.

However, the influences of background flow shear on the spatial structure, propagation velocity and wave width of solitary Rossby waves remain absent. The objectives of this article are to elucidate the background flow horizontal shear effects on Rossby waves in a nonlinear regime, and to interpret those observed characteristics of isolated anomalies that classical theory cannot explain.

## Theory and Methodology

We consider the following nondimensional reduced-gravity quasi-geostrophic potential vorticity equation^33^:

1 $$\frac{\partial }{\partial t}({\nabla }^{2}\psi -F\psi )+J(\psi ,{\nabla }^{2}\psi )+\beta \frac{\partial \psi }{\partial x}=0,$$

where $F={L}^{2}/{R}_{d}^{2}$, *L* is a characteristic length scale of the motion, *R*_*d*_ is the Rossby deformation radius, *ψ* is the stream function, *β* = β_*_L^2^/*U* is the gradient of the Coriolis parameter, and *U* is the characteristic horizontal velocity scale of the motion.

The horizontal velocity can be obtained from

$${u}_{g}=-\,\frac{\partial \psi }{\partial y},\,\,{v}_{g}=\frac{\partial \psi }{\partial x}.$$

In this paper, the dimensional variables are denoted by an asterisk, for example y_*_ = *yL*, where *L* represents domain size. The boundary condition is

2 $$\frac{\partial \psi }{\partial x}=0\,{\rm{at}}\,y=0,\,1.$$

The background stream function has the form

3 $${\phi }_{0}(y)=-\,\int u(y)dy.$$

We take the total stream function *ψ* as a disturbance stream *εϕ* pre-imposed on the zonal flow *u*:

4 $$\psi ={\phi }_{0}(y)+\varepsilon \phi =-\,\int u(y)dy+\varepsilon \phi .$$

where *ε* represents the Rossby number, which is a small parameter. Substitution of (4) into (1) yields

5 $$\frac{\partial }{\partial t}({\nabla }^{2}\phi -F\phi )+u\frac{\partial }{\partial x}{\nabla }^{2}\phi +(\beta -{u}_{yy})\frac{\partial \phi }{\partial x}+\varepsilon J(\phi ,{\nabla }^{2}\phi )=0.$$

In order to balance the nonlinearities and dispersions, we introduce the following slow space and time scales^15^:

6 $$X={\varepsilon }^{1/2}(x-{c}_{0}t),T={\varepsilon }^{3/2}t,$$

where *c*_0_ is the linear Rossby wave phase speed, which can be determined by solving following eigenvalue problem. Using the multiple scale method, we write the disturbance stream function as

$$\phi ={\phi }_{1}+\varepsilon {\phi }_{2}+\ldots ,$$

where *ϕ*_1_ = *A*(*X*, *T*)*ψ*_1_(*y*), *A*(*X*, *T*) is the amplitude, and *ψ*_1_(*y*) is the meridional structure of the waves. After a series of manipulations (see Supplementary Equations 1), we obtain the KdV equation^21^ for the amplitude *A*(*X*, *T*)

7 $$\frac{\partial A}{\partial T}+{a}_{1}A\frac{\partial A}{\partial X}+{a}_{2}\frac{{\partial }^{3}A}{\partial {X}^{3}}=0,$$

and the eigenvalue equations for *ψ*_1_(*y*)

8 $$\frac{{\partial }^{2}{\psi }_{1}}{\partial {y}^{2}}+\frac{\beta -{u}_{yy}+F{c}_{0}}{u-{c}_{0}}{\psi }_{1}=0,\,{\psi }_{1}(y=0,1)=0,$$

where

9 $$\begin{array}{rcl}{a}_{1} & = & \frac{1}{{a}_{0}}{\int }_{0}^{1}\,\frac{{\psi }_{1}^{3}}{u-{c}_{0}}{(\frac{\beta -{u}_{yy}+F{c}_{0}}{u-{c}_{0}})}_{y}dy,\\ {a}_{2} & = & -\frac{1}{{a}_{0}}{\int }_{0}^{1}\,{\psi }_{1}^{2}dy,\\ {a}_{0} & = & {\int }_{0}^{1}\,\frac{\beta -{u}_{yy}+Fu}{{(u-{c}_{0})}^{2}}{\psi }_{1}^{2}dy.\end{array}$$

Benney^20^, Redekopp^27^, Yamagata^22^, and Warn^23^ also obtained the same KdV equation as equation (7) and pointed out that the background flow shear is a necessary condition for the existence of solitary waves. However, the influences of background flow shear on the spatial structure, propagation velocity and wave width of solitary Rossby waves have not been studied.

## Background Flow Effects

If there is no shear in the background flow, the solution of the eigenvalue equations (8) can be obtained analytically:

$$\begin{array}{ccc}{\psi }_{1} & = & \sin \,n\pi y,n=1,2,\mathrm{...},\\ {c}_{0} & = & u-\frac{\beta +Fu}{{(n\pi )}^{2}+F},\end{array}$$

where *c*_0_ is the phase speed of the linear long mode *n* Rossby wave (*k*→0, where k represents the wavenumber in the horizontal direction). The governing equation of the amplitude (7) can be reduced to:

10 $$\frac{\partial A}{\partial T}+{a}_{2}\frac{{\partial }^{3}A}{\partial {X}^{3}}=0,{a}_{2}=-\frac{\beta +Fu}{{({n}^{2}{\pi }^{2}+F)}^{2}},$$

which is a linear dispersion equation, and it can be solved using an ordinary Fourier series. In this case, Rossby waves have north–south symmetry and their shape can be convex or concave.

If there is shear in the background flow, *u*_*y*_ ≠ 0, and as *u* is function of variable *y*, it is difficult to obtain the analytic solution of eigenvalue equations (8); but it can be solved using numerical methods or asymptotic analytical method. Analytic expressions can more easily reveal the effect of background flow on Rossby waves, so the asymptotic analytical method is used to solve the eigenvalue equations. From Fig. 1a, the obvious feature of the flow field in the subtropical countercurrent zone is meridional shear of zonal current. In the model, we consider weak shear flow

11 $$u(y)={u}_{0}+\delta y,$$

where *δ* << 1, which can capture the main characteristics of the background flow. For other forms of background flow, it can also be solved using the asymptotic analytical method. In order to solve the eigenvalue equations (8), we assume a regular perturbation series expansion and obtain (see Supplementary Equations 2 for the method in detail):

12 $$\{\begin{array}{rcl}{\psi }_{1} & = & \sin \,n\pi y+\delta \frac{1}{4}\frac{n\pi }{{u}_{0}-\overline{{c}_{0}}}[(y-{y}^{2})cosn\pi y+\frac{y}{n\pi }sinn\pi y]+O({\delta }^{2})\\ {c}_{0} & = & {u}_{0}-\frac{\beta +F{u}_{0}}{{(n\pi )}^{2}+F}+\delta \frac{1}{2}\frac{{(n\pi )}^{2}}{{(n\pi )}^{2}+F}+O({\delta }^{2})\\ \overline{{c}_{0}} & = & {u}_{0}-\frac{\beta +F{u}_{0}}{{(n\pi )}^{2}+F}.\end{array}$$

These equations imply that the meridional structure of solitary wave (eddy) is asymmetry because of the basic flow shear.

Substituting (11) and (12) into (9) and omitting the *O*(*δ*^2^) term gives:

13 $$\begin{array}{rcl}{a}_{1} & = & -\frac{2}{{a}_{0}}\frac{(1-{(-1)}^{n})n\pi }{3}\frac{{({(n\pi )}^{2}+F)}^{2}}{{(\beta +F{u}_{0})}^{2}}\delta ,\\ {a}_{2} & = & -\frac{1}{{a}_{0}}(\frac{1}{2}+\frac{1}{8}\frac{{n}^{2}{\pi }^{2}+F}{\beta +F{u}_{0}}\delta ),\\ {a}_{0} & = & \frac{1}{2}\frac{{({(n\pi )}^{2}+F)}^{2}}{\beta +F{u}_{0}}-\frac{1}{8}\frac{(F-{n}^{2}{\pi }^{2}){({(n\pi )}^{2}+F)}^{2}}{{(\beta +F{u}_{0})}^{2}}\delta .\end{array}$$

Using Jacobi elliptic function expansion methods and equation (6), the cnoidal waves solution of (7) is

14 $$A(x,t)={A}_{0}{{\rm{cn}}}^{2}\{{\varepsilon }^{\tfrac{1}{2}}\sqrt{\frac{{A}_{0}{a}_{1}}{12{a}_{2}{m}^{2}}}[x-({c}_{0}+\frac{{A}_{0}{a}_{1}}{3}(2-\frac{1}{{m}^{2}})\varepsilon )t-\frac{{X}_{0}}{\sqrt{\varepsilon }}],m\},$$

where *m* represents the modulus of Jacobi elliptic function, *A*_0_ is the amplitude of the waves, ${\varepsilon }^{-\tfrac{1}{2}}\sqrt{\frac{12{a}_{2}{m}^{2}}{{A}_{0}{a}_{1}}}$ represents the width of the waves, and ${c}_{0}+\frac{{A}_{0}{a}_{1}}{3}(2-\frac{1}{{m}^{2}})\varepsilon$ is the phase speed of the waves. When *m*→1, cnoidal waves solution (14) reduced to solitary waves solution, which is a nondispersive wave, and it implies the wave maintains its shape while propagating at a constant velocity. The nondispersive characteristic of SSH anomaly has been confirmed by Chelton and Schlax^32^ and Chelton *et al*.^12^, because the eddy-like structures retain their shapes and the energy at every wavenumber propagates at the same speed. From equation (14), the width of the solitary Rossby wave is:

15 $$w={\varepsilon }^{-\tfrac{1}{2}}\sqrt{\frac{12{a}_{2}{m}^{2}}{{A}_{0}{a}_{1}}}\approx \frac{3m|\beta +F{u}_{0}|}{({n}^{2}{\pi }^{2}+F)\sqrt{\varepsilon }}\frac{1}{\sqrt{(1-{(-1)}^{n})n\pi }\sqrt{{A}_{0}\delta }},$$

which implies the width of the solitary wave is inversely proportional to the flow shear strength |*δ*|, and that (*A*_0_*δ*) must be positive to keep this width physical meaningful. In other words, if the background flow shear is positive (*δ* > 0), *A*_0_ must be positive too. Positive *A*_0_ means there are anticyclonic solitary Rossby waves (*A*_0_ > 0, *n* = 1). On the other hand, if it is negative (*δ* < 0), there are cyclonic solitary Rossby waves (*A*_0_ < 0, *n* = 1). When *n*→∞, the width *w* will tend to be zero and the sea surface will seem like flat that corresponds to the background geostrophic current. When *n* = 1, the solitary wave shows the largest width. When *ε*→0(the current is absolutely in geostrophy), the *w*→∞ and it means that given a constant energy of solitary wave, the amplitude will be negligible and the solution is the background geostrophic current.

The phase speed of the solitary Rossby waves is

16 $$c={c}_{0}+\frac{{A}_{0}{a}_{1}}{3}(2-\frac{1}{{m}^{2}})\varepsilon .$$

It is important to note that the phase speed of solitary Rossby wave proportional to its amplitude and could be faster or slower than the classical Rossby wave phase speed. When $m > 1/\sqrt{2}$, it is faster than the classic Rossby wave phase speed *c*_0_. This feature agrees with the observations that large-amplitude solitary Rossby waves propagate faster than small-amplitude waves^1^. A notable feature of solitary Rossby waves that distinguishes them from linear Rossby waves is that their speed is dependent on amplitude. Furthermore, this solution shows another kind of solitary Rossby wave whose phase speed is slower than the classic Rossby phase speed. This solution remains to be verified by observations.

## Results and Discussion

From the first equation of (13), *n* must be an odd number, otherwise, a solitary Rossby wave cannot exist. The present analysis takes *n* = 1 and it focuses on latitude 23°N in the North Pacific. At this latitude, the typical values of parameters for oceanic solitary Rossby waves are β_*_ = 2.1 × 10^−11^ *m*^−1^*s*^−1^, *L* = 1.6 × 10^5^ *m*, *ε* = 0.2, *U* = 0.045 *ms*^−1^ and *R*_*d*_ = 5 × 10^4^ *m*^34^. We take |*A*_0_| = 1 and u_*_ as follows:

17 $${u}_{\ast }=Uu=-U+U\delta {y}_{\ast }/L=-0.045(1-\delta {y}_{\ast }/L),$$

where u_*_ is smaller than the linear long Rossby wave phase velocity ${\beta }_{\ast }{R}_{d}^{2}\approx 0.0525m{s}^{-1}$.

### Background flow shear effects on meridional structure

**Figure 3:**
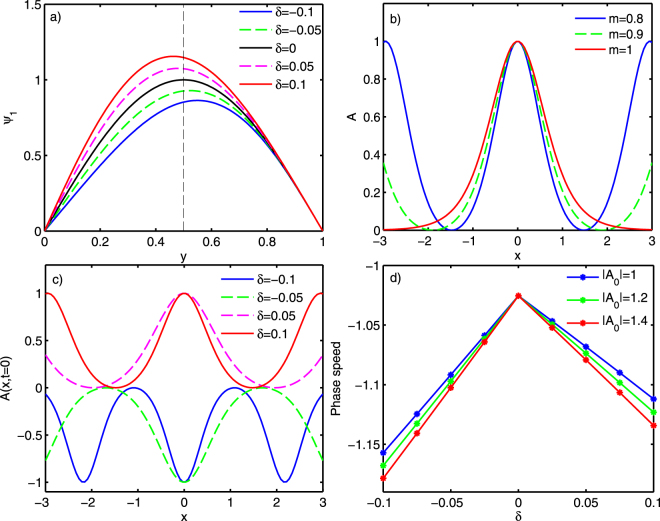
Characteristics of solitary Rossby waves when *n* = 1: (**a**) Meridional structure *ψ*_1_ with different values of *δ*. (**b**) Amplitude *A* with different values of *m*(*δ* = 0.1). (**c**) Amplitude *A* with different values of *δ*(*m* = 0.8). (**d**) Phase speed of Rossby waves with different values of *A*_0_ and *δ*.

The meridional structure of Rossby waves can be determined from (12). From Fig. 3a, we see that when *δ* < 0, the meridional structure tilts northward and when *δ* > 0, the meridional structure tilts southward. When *δ* = 0, which reduces to linear Rossby waves, the meridional structure has north–south symmetry.

### Background flow shear effects on amplitude and width

The amplitude *A* in (14) of Rossby waves with different values of *m* (*δ* = 0.1) and *δ* (*m* = 0.8), when *X*_0_ = 0, *t* = 0, and |*A*_0_| = 1, is shown in Fig. 3b,c. Here, *m* represents the modulus of the cnoidal waves (14), which is a free parameter ranged from 0 to 1, and we assume *m* = 0.8. If *m*→0, the cnoidal waves reduce to cosine waves and if *m*→1, the cnoidal waves reduce to solitary waves. The value of *m* essentially has no significant effect on our conclusions. When the flow shear is negative (*δ* < 0), cyclonic solitary Rossby waves occurs (*A* < 0). When the flow shear is positive (*δ* > 0), anticyclonic solitary Rossby waves occurs (*A*>0). When *δ* = 0, the model degenerates into the linear model and either anticyclonic or cyclonic solitary Rossby waves exist (the amplitude can be arbitrary).

In the width solution, it is important to note that the bigger the *A*_0_ is, the smaller the width is. It means that this solution permits strong SSH anomalies with a small diameter. Moreover, it is interesting to note that the width of the solitary wave is proportional to the gradient of the planetary vorticity *β* and *u*_0_. Thus, the wave width tends to increases equatorward. However, this equation is not applicable to equator because geostrophic balance in the lowest order needed by equation (1) will fail in the equator. In addition, the width *w* is larger when *u*_0_ is strong. It is easy to understand that the solitary wave could easily get energy from the strong *u*_0_ by barotropic instability. The width of the solitary Rossby waves is also related to the flow shear; the weaker the flow shear, the larger the width of the solitary Rossby waves. Then, this solution is physically meaningful.

### Background flow shear and amplitude effects on phase speed

Figure 3d depicts the background flow shear and amplitude effects on phase speed using (16), where the phase speeds are scaled with *c*_0_. For a given flow shear (*δ* ≠ 0), the phase speed is proportional to the amplitude (|*A*_0_|), and the greater the shear, the more significant the effect of amplitude on the phase speed. If *δ* = 0, the model degenerates into the linear model and the amplitude has no effect on phase speed. For a given amplitude, the phase speed is proportional to the intensity of the background flow shear (|*δ*|), and the minimum value of phase speed is obtained when *δ* = 0, which is exactly the result of the linear model. It is worth noting the small difference in phase speed when *δ* takes a different sign. This occurs because the flow shear changes the background flow, which further changes the phase speed because of the Doppler shift. However, the flow shear effect is much smaller than the amplitude effect on the phase speed.

**Figure 4:**
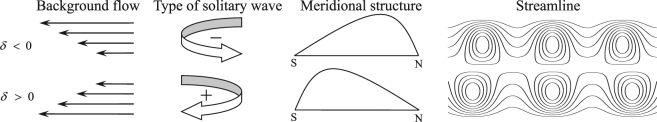
Background flow shear effects on Rossby waves.

The above discussion shows the background flow shear has significant impact on the Rossby waves. Consideration of the nonlinear effects shows that solitary Rossby waves exist with properties that are quite different from classic Rossby waves but consistent with satellite observations. If the background flow shear is positive (negative), anticyclonic (cyclonic) solitary Rossby waves exist and their meridional structure is tilted southward (northward), which is consistent with the observations that the eddies propagate westward at approximately the phase speed of linear baroclinic Rossby waves, and cyclonic (anticyclonic) eddies deviate slightly poleward (equatorward) with respect to the latitude circle^11,12^. In addition, the background flow shear also contributes to the band-like eddy polarity pattern in the STCC zone (see Fig. 2). Furthermore, their phase speed is greater than that predicted by classic Rossby wave theory; larger amplitude solitary Rossby waves have faster phase speeds^1,32^. The increase of phase speed is not very large (~10%), because of the weak shear itself. A stronger shear is expected to correspond to more significant modulation of wave properties, but this will need to be confirmed by a separate study. Using sea-surface height fields constructed from the merged TOPEX/Poseidon and ERS-1/2 altimeter datasets, Chelton *et al*.^11^ investigated global ocean mesoscale eddies in detail. The propagation speeds and directions of the observed eddies are consistent with the theories for nonlinear dynamics, which predict that eddies should propagate westward with little meridional deflection at the phase speeds of nondispersive baroclinic Rossby waves^13^. The poleward (equatorward) movement of westward propagating cyclonic (anticyclonic) eddies are expected from the combination of the *β* effect and self advection. The observed weak dispersion in wavenumber frequency spectra of SSH also confirm the property of nondispersion^32^, because the eddies retain their shapes as they propagate the energy at every wavenumber propagates at the same speed. The eddies generation mechanism is most likely due to the instability of the background currents. The width of solitary wave is inversely related to the background current shear strength. If the shear is too strong, it will produce sub-mesoscale eddies, such as the distribution of eddies across the Kuroshio in the East China Sea^35^. The asymmetrically distribution of eddies across the Kuroshio in the East China Sea (predominant cyclonic eddies are on the western sides of Kuroshio and anticyclonic eddies are on the eastern sides of Kuroshio) is also related to the different horizontal velocity shear on both sides of the Kuroshio axis^35^. These effects of background flow shear on solitary Rossby waves are summarized in Fig. 4.

### Structural characteristics of solitary Rossby waves when *n* = 3

**Figure 5:**
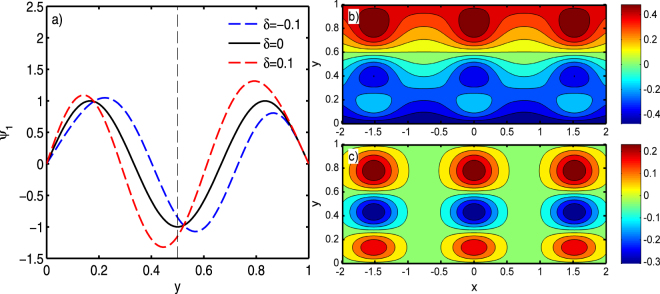
Characteristics of solitary Rossby waves when *n* = 3: (**a**) Meridional structure *ψ*_1_ with different values of *δ*. Streamlines of solitary Rossby waves (**b**) with background flow and (**c**) without background flow when *δ* = 0.1.

In this section, we choose *L* = 5 × 10^5^ *m*, *n* = 3, and *δ* = 0.1, and the other symbols have the same values as discussed above. The background flow shear effects on amplitude, width, and phase speed are consistent with the conclusions derived when *n* = 1; the obvious difference is the meridional structure *ψ*_1_. The meridional structures and streamlines of solitary Rossby waves are illustrated in Fig. 5. When *δ* = 0.1, the meridional structure tilts southward, and both the amplitude and the width are larger in the north. In contrast, when *δ* = −0.1, the meridional structure tilts northward, and both the amplitude and the width are larger in the south. It is reasonable that the meridional shear of zonal current determine the southward or northward tilting.

## Summary

In this paper, by employing perturbation expansions and scale transformations of the time and space method, an asymptotic solution of the potential vorticity equation was derived. For a specific background zonal flow *u* = *u*_0_ +*δy*, the effects of flow shear on the meridional structure, amplitude, width, and phase speed of solitary Rossby waves were studied.

Background flow shear is required for the existence of solitary Rossby waves. Cyclonic (anticyclonic) solitary Rossby waves exist in a cyclonic (anticyclonic) mean flow, and their meridional structure tilts northward (southward). The phase speed of solitary Rossby waves is proportional to the amplitude, and the greater the flow shear, the more significant the effect of amplitude on phase speed. The phase speed is also proportional to the intensity of the flow shear (|*δ*|). Solitary Rossby waves are non-dispersive waves, and the width of solitary Rossby waves is inversely proportional to the intensity of the flow shear (|*δ*|). Chelton *et al*.^1^ pointed out that the standard theory for free, linear Rossby waves is an incomplete description of the observed waves. Observed characteristics of isolated anomalies were found to be broadly consistent with our nonlinear quasi-geostrophic theory, which suggest the importance of nonlinear dynamics. Based on the analytical solution, the background flow shear effect can be used to explain the band-like distribution of mesoscae eddies in the subtropical counter current zone.

In the theoretical analysis, because *c*_0_ < 0, for westward mean flows (*u* < 0) equation may imply instability due to singular behavior near a potential critical layer, i.e. a latitude where *u* = *c*_0_. However, for eastward currents (*u* > 0), this singular behavior is absent. Therefore the solitary wave solution is nearly absent in area where the zonal current flows eastward, while solitary waves (eddies) can be expected to be found in the westward flowing zonal current area. The solution is only applicable to the area with a weak zonal shear, and strongly sheared flows may exhibit different properties. Flow with a strong horizontal shear will also be subject to barotropic instability, which can further modify the solution. Furthermore, it is especially favorable in the westward flowing zonal current area. In the STCC zone, solitary eddies mainly concentrate in the areas with weak background flow (north of 20°N), while the number of solitary eddies in the area with strong westward flow (south of 20°N) is less (see Fig. 1). In addition, baroclinic instability also plays an important role in the modulation of the solitary waves (eddies)^30^. Furthermore, our solution shows another kind of solitary Rossby wave whose phase speed is slower than the classic Rossby wave phase speed. This solution remains to be examined by observations in the future. In addition to the STCC zone, our theoretical results are also applicable to other similar ocean areas in the world.

### Data Availability

The ADT and SLA data were generated by DUACS and distributed by AVISO (ftp://ftp.aviso.altimetry.fr/).

## Electronic supplementary material

Supplementary Information
