# Supplementary material for: One possible mechanism for eddy distribution in zonal current with meridional shear
Source: Sci Rep. 2018 Jul 4;8:10106. doi: 10.1038/s41598-018-28465-z (PMC6031702; doi:10.1038/s41598-018-28465-z)
Supplement: Supplementary file 1 — Supplementary Information [file 41598_2018_28465_MOESM1_ESM.docx]

**Supplementary Information for**

**One possible mechanism for eddy distribution in zonal current with meridional shear**

YunLong Shi^1,2,3^, DeZhou Yang^2,4^, XingRu Feng^2^, JiFeng Qi^2^, HongWei Yang^5^ and BaoShu Yin^2,3,4^

^1^School of Marine Sciences, Nanjing University of Information Science and Technology, Nanjing, China. ^2^Key Laboratory of Ocean Circulation and Waves, Institute of Oceanology, Chinese Academy of Sciences, Qingdao, China. ^3^University of Chinese Academy of Sciences, Beijing, China. ^4^Function Laboratory for Ocean Dynamics and Climate, Qingdao National Laboratory for Marine Science and Technology, Qingdao, China. ^5^College of Mathematics and System Science, Shandong University of Science and Technology, Qingdao, China.

Corresponding author: DeZhou Yang (yangdezhou@qdio.ac.cn)

Supplementary Equations 1

In this Supplementary Equations, we give the detailed derivation of (7).

Substituting (6) into (5) leads to the following:

(S1)

Using the multiple scale method, the disturbance stream function has the form of

.

By collecting terms of order $O(\varepsilon^{0})$, we have the following equation in

(S2)

The term of order $O(\varepsilon)$ gives:

(S3)

We take the solution of (S2) in the form

(S4)

By substituting (S4) into (S2) , we obtain the following eigenvalue equation for $\psi_{1}(y)$:

$\frac{\partial^{2}\psi_{1}}{\partial y^{2}}+\frac{\beta-u_{yy}+Fc_{0}}{u-c_{0}}\psi_{1}=0,$ (S5)

$\psi_{1}=0 \text{at} y=0,1.$ (S6)

Solving (S5) with boundary condition (S6) means the eigenvalue $c_{0}$and meridional structure $\psi_{1}$ can be determined.

To derive the amplitude $A(X,T)$, we sought the solution of the higher order problem (S3). Similar to (S4), we assume is in the form

(S7)

Multiplying both sides of (S3) by $\psi_{1}(y)/(u-c_{0})$, and integrating with respect to $y$ over $[0 1]$, we have

$\frac{\partial A}{\partial T}+a_{1}A\frac{\partial A}{\partial X}+a_{2}\frac{\partial^{3}A}{\partial X^{3}}=0,$ (S8)

which is the KdV equation, where

$\begin{matrix} a_{1}= \frac{1}{a_{0}}\int_{0}^{1} \frac{\psi_{1}^{3}}{u-c_{0}}\left(\frac{\beta-u_{yy}+Fc_{0}}{u-c_{0}}\right)_{y} dy, \\ a_{2}= -\frac{1}{a_{0}}\int_{0}^{1} \psi_{1}^{2}dy, \\ a_{0}= \int_{0}^{1} \frac{\beta-u_{yy}+Fu}{(u-c_{0})^{2}}\psi_{1}^{2}dy. \end{matrix}$ (S9)

Using Jacobi elliptic function expansion methods, the cnoidal waves solution of (S8) is

$A(X,T)=A_{0}\text{cn}^{2} \left\{\sqrt{\frac{A_{0}a_{1}}{12a_{2}m^{2}}}\left[X-\frac{A_{0}a_{1}}{3}\left(2-\frac{1}{m^{2}}\right)T\right],m\right\},$ (S10)

Supplementary Equations 2

In this Supplementary Equations, we give the detailed derivation of (12).

In order to solve the eigenvalue equations (8), we assume a regular perturbation series expansion

(S11)

Substituting (11) and (S11) into (8) gives:

(S12)

(S13)

Taking the solutions of (S12) as

(S14)

and substituting (S14) into (S13), we obtain from the solvability condition of (S13):

(S15)

Then, we have

$\left\{\begin{matrix} \psi_{1}= \sin n\pi y+\delta\frac{1}{4}\frac{n\pi}{u_{0}-\overline{c_{0}}}\left[\left(y-y^{2}\right)\cos n\pi y+\frac{y}{n\pi}\sin n\pi y\right]+O(\delta^{2}) \\ c_{0}= u_{0}-\frac{\beta+Fu_{0}}{(n\pi)^{2}+F}+\delta\frac{1}{2}\frac{(n\pi)^{2}}{(n\pi)^{2}+F}+O(\delta^{2}). \end{matrix}\right.$ (S16)
